# Characteristics of *Escherichia coli* ST131 strains isolated from dogs and cats with urinary tract infections in a teaching hospital in Taiwan

**DOI:** 10.1371/journal.pone.0350088

**Published:** 2026-05-22

**Authors:** Shu-Xian Lin, Kuang-Sheng Yeh

**Affiliations:** 1 Graduate Institute of Veterinary Medicine, School of Veterinary Medicine, National Taiwan University, Taipei, Taiwan; 2 Department of Veterinary Medicine, School of Veterinary Medicine, National Taiwan University, Taipei, Taiwan; Universidad San Francisco de Quito, ECUADOR

## Abstract

*Escherichia coli* sequence type (ST) 131 is a globally disseminated multidrug-resistant clone that poses a substantial public health threat. This study investigated the prevalence, virulence characteristics, and antimicrobial resistance profiles of ST131 among companion-animal *E. coli* isolates. A total of 400 *E. coli* isolates obtained from dogs and cats diagnosed with urinary tract infections at the National Taiwan University Veterinary Hospital between 2011 and 2019 were analyzed. Phylogenetic grouping identified 192 isolates (48.0%) belonging to phylogroup B2, which is strongly associated with ST131. Among these, 26 isolates (13.5%) were confirmed as ST131 by single-nucleotide polymorphism screening and multilocus sequence typing, and 19 isolates were selected for detailed characterization. Virulence gene analysis by multiplex PCR showed that *fyuA* (100.0%), *traT* (94.7%), *iutA* (89.5%), and *kpsMT* II (84.2%) were the most prevalent genes. Antimicrobial susceptibility testing demonstrated universal susceptibility to meropenem, whereas all isolates were resistant to amoxicillin and ampicillin. Six canine-derived isolates produced extended-spectrum β-lactamases (ESBLs) or AmpC β-lactamases. Among ESBL producers, *bla* gene groups *bla*_CTX-M-1_, *bla*_CTX-M-9_, and *bla*_TEM_ were detected, whereas *bla*_CMY-178_ was the only AmpC gene identified. Conjugation experiments demonstrated transferability for most *bla* genes, except *bla*_TEM-102_, *bla*_TEM-12_, and *bla*_CTX-M-55_. Plasmid replicon typing revealed IncF (100%) and IncB/O (69%) as the predominant plasmid groups. Only two O-antigen types, O25b and O16, were detected among ST131 isolates, which carried *fimH* variants *fimH22*, *fimH27*, *fimH30*, or *fimH41* subclones. These findings demonstrate the circulation of virulent and multidrug-resistant ST131 strains in companion animals in Taiwan, highlighting their potential relevance within a One Health context.

## Introduction

*E. coli* is a common commensal bacterium of the intestinal tract but also an important opportunistic pathogen capable of causing both intestinal and extraintestinal infections [1]*.* While many strains are harmless members of the normal microbiota, pathogenic strains may acquire virulence determinants through horizontal gene transfer mediated by plasmids, bacteriophages, or chromosomal integration. Pathogenic *E. coli* are broadly classified into intestinal pathogenic *E. coli*, which cause diarrheal disease, and extraintestinal pathogenic *E. coli* (ExPEC), which cause infections outside the gastrointestinal tract [2]. ExPEC is a major cause of urinary tract infections (UTIs), septicemia, and meningitis, resulting in substantial clinical and economic burdens worldwide. In the United States alone, ExPEC infections are estimated to account for nearly USD 2 billion annually in direct medical costs [3]. The growing prevalence of antimicrobial resistance has further complicated treatment options. For example, trimethoprim–sulfamethoxazole, once widely used for UTIs, has increasingly been replaced by fluoroquinolones because of resistance; however, extensive fluoroquinolone use has subsequently driven the emergence of fluoroquinolone-resistant *E. coli* strains [4].

In addition to antimicrobial resistance, ExPEC strains possess numerous virulence factors that enhance their pathogenic potential [5]. Among these, *E. coli* sequence type (ST)131 has attracted global attention since its first recognition in North America, Europe, and Asia in 2008 [6–8]. ST131 is characterized by high virulence, multidrug resistance, and widespread international dissemination, posing important threats to both human and animal health [9]. *E. coli* ST131 is an important cause of UTIs in both humans and companion animals, particularly dogs and cats [10]. In 2009, ST131 carrying *bla*_CTX-M-15_ and fluoroquinolone resistance determinants including *qnrB2* and *aac(6’)-Ib-cr* was reported from canine urine samples in Portugal [11]. Close contact between humans and pets may facilitate bidirectional transmission [11], and previous studies have shown that cohabiting dogs and cats can harbor indistinguishable ST131 strains [12]. Furthermore, ST131 has also been identified in livestock, wildlife, and environmental reservoirs, emphasizing its relevance within a One Health framework [9].

Although previous studies have reported extended-spectrum β-lactamase-producing *E. coli* from companion animals in Taiwan [13–15], detailed molecular characterization of ST131 isolates from veterinary clinical cases remains limited. Such information is important for understanding local epidemiology, tracking the dissemination of antimicrobial resistance, and supporting antimicrobial stewardship. Therefore, this study aimed to determine the prevalence and molecular characteristics of *E. coli* ST131 isolated from dogs and cats with urinary tract infections in a veterinary teaching hospital in Taiwan, including their antimicrobial susceptibility, virulence gene repertoire, β-lactamase genotypes, plasmid content, serotype–*fimH* associations, and transferability of resistance determinants.

## Materials and methods

### Sample collection

A total of 400 *E. coli* isolates were recovered from urine samples of dogs and cats diagnosed with UTIs at the National Taiwan University Veterinary Hospital between 2011 and 2019. Species identification was performed using the VITEK 2 Compact automated system (bioMérieux, Marcy-l'Étoile, France) with GN identification cards. All isolates were preserved in Microbank System cryovials (Pro-Lab Diagnostics, Richmond Hill, Ontario, Canada) and stored at −80 °C until analysis. The clinical samples used in this study were collected as part of routine diagnostic and therapeutic procedures. No animals were sampled specifically for research purposes; therefore, formal approval from an Institutional Animal Care and Use Committee (IACUC) was not required.

### *E. coli* phylogenetic grouping

Heat-extracted lysates from all 400 isolates were prepared as previously described by Shaheen et al. [16] and used as DNA templates for polymerase chain reaction (PCR). Phylogenetic grouping was performed according to the revised Clermont quadruplex PCR method [17], which classifies *E*. *coli* into phylogroups A, B1, B2, C, D, E, F, and clade I based on the presence or absence of specific amplicons. Isolates showing the marker combinations (−, + , + , −), (−, + , + , +), or (−, + , − , +) for *arpA*, *chuA*, *yjaA*, and Tsp.E4.C2 were assigned to phylogroup B2. Primer sequences are listed in S1 Table.

### Screening and confirmation of *E. coli* ST131

Among the phylogroup B2 isolates, screening for ST131 was performed using previously described single-nucleotide polymorphism (SNP)-based markers in the *mdh* and *gyrB* genes. Specifically, ST131-associated SNPs included C288T and C525T in *mdh*, and C621T, C729T, and T735C in *gyrB*. Target gene fragments were amplified by PCR and sequenced. The resulting sequences were compared with reference sequences available in the National Center for Biotechnology Information (NCBI) database [18]. Putative ST131 isolates were further confirmed by multilocus sequence typing (MLST), which was performed using seven housekeeping genes as previously described [19]. PCR products were sequenced commercially, and allele profiles were analyzed using the PubMLST (https://pubmlst.org/) and EnteroBase databases (http://enterobase.warwick.ac.uk/) for sequence type assignment. Primer sequences are listed in S2 Table.

### Virulence gene detection

Virulence-associated genes were detected by multiplex PCR using the method of Johnson and Stell [20]. Twelve genes commonly associated with uropathogenic *E. coli* were examined, including adhesin genes (*papAH*, *sfa*/*focDE*, *focG*), siderophore genes (*iutA*, *fyuA*), toxin genes (*cnf1*, *hlyA*), capsule-associated genes (*K1*, *kpsMT* II, *K5*), and additional virulence-associated genes (*ibeA* and *traT*). Primer sequences are shown in S3 Table.

### Antimicrobial Susceptibility Testing (AST)

Antimicrobial susceptibility testing was performed using the minimum inhibitory concentration (MIC) method according to Clinical and Laboratory Standards Institute (CLSI) guidelines [21]. Fifteen antimicrobial agents were evaluated: ceftiofur, cefotaxime (CTX), ceftazidime (CAZ), meropenem, amoxicillin, ampicillin, colistin, amikacin, kanamycin, gentamicin, doxycycline, tetracycline, ciprofloxacin, enrofloxacin, and trimethoprim–sulfamethoxazole.

### Screening and confirmation of ESBL-producing *E. coli* ST131

ST131 isolates were initially screened for extended-spectrum β-lactamase (ESBL) production using CHROMagar ESBL selective medium (CHROMagar, Paris, France). Presumptive ESBL-positive isolates were further confirmed phenotypically using the CLSI combination disk diffusion method with ceftazidime, ceftazidime–clavulanic acid, cefotaxime, and cefotaxime–clavulanic acid disks. ESBL production was confirmed when the inhibition zone diameter increased by ≥5 mm in the presence of clavulanic acid. *Klebsiella pneumoniae* ATCC 700603 and *E. coli* ATCC 25922 were used as positive and negative controls, respectively.

### AmpC-β-lactamase phenotype test

AmpC β-lactamase production was evaluated using cefotetan/cefotetan-cloxacillin gradient strips according to the method of Polsfuss et al. [22]. A ratio of the MIC of cefotetan alone to that of cefotetan plus cloxacillin of ≥8 was interpreted as positive for AmpC β-lactamase production. *K. pneumoniae* ATCC 700603 and strains previously confirmed as AmpC-β-lactamase producers served as the negative and positive controls, respectively.

### Detection of β-lactamase genes of *E. coli* ST131

ESBL- or AmpC-producing ST131 isolates were screened by PCR for common β-lactamase genes. Target genes included *bla*_TEM_, *bla*_SHV_, *bla*_CTX-M_ groups *(bla*_CTX-M-1_, _−2_, _−8_, _−9_, and _−25_) [23–27], and plasmid-mediated AmpC genes *(bla*_CIT_, *bla*_DHA_, *bla*_MOX_, *bla*_CMY_, *bla*_EBC_, and *bla*_FOX_) [28]. Amplicons of the expected size were sequenced, and sequences were compared with entries in the Beta-Lactamase Database and NCBI databases (https://www.ncbi.nlm.nih.gov/pathogens/beta-lactamase-data-resources/). The primers used for detecting β-lactamase genes are listed in S4 Table.

### O-antigen and *fimH* subclone typing

O-antigen typing focused on the two serotypes most commonly associated with ST131, O25b and O16. Multiplex PCR targeting *pabB* (O25b) and *trpA* (O16) was performed as previously described [29,30]. *fimH* subclone typing was conducted by multiplex PCR to detect *fimH22*, *fimH27*, *fimH30*, *fimH30Rx*, *fimH35*, and *fimH41* [31]. The primers used for these assays are listed in S5 Table.

### Plasmid replicon typing

Plasmid replicon types were determined by multiplex PCR using the method of Johnson et al [32]. Detected plasmids were classified according to incompatibility (Inc) groups. S6 Table lists the primers for plasmid replicon typing.

### Conjugation test

Conjugation experiments were performed to evaluate horizontal transfer of β-lactamase genes. ST131 isolates were used as donor strains, and sodium azide-resistant *E. coli* J53 (ATCC BAA-2730) served as the recipient strain. Donor and recipient cultures were mixed and incubated overnight, and transconjugants were selected on Mueller–Hinton agar containing cefotaxime (2 mg/L) and sodium azide (150 mg/L) [33]. Putative transconjugants were confirmed by PCR for the corresponding resistance genes.

### Statistical analysis

This study primarily used descriptive statistics. The prevalence of phylogenetic groups, ST131 isolates, virulence genes, antimicrobial resistance phenotypes, β-lactamase genes, O-antigen types, *fimH* subclones, and plasmid replicon types was summarized as counts and percentages. No inferential statistical comparisons were performed because the study was designed to characterize the molecular features of ST131 isolates rather than to test predefined hypotheses between groups.

## Results

### *E. coli* phylogenetic grouping

A total of 400 *E. coli* isolates were recovered from urine samples of dogs and cats diagnosed with urinary tract infections at the National Taiwan University Veterinary Hospital between December 6, 2011, and November 13, 2019. Of these, 314 isolates (78.5%) were obtained from dogs and 86 (21.5%) from cats. Phylogenetic grouping showed that group B2 was the most prevalent group (192/400, 48.0%), including 144 isolates from dogs (75.0%) and 48 from cats (25.0%). The remaining isolates were assigned to group E (52/400, 13.0%), group B1 (39/400, 9.8%), group A (12/400, 3.0%), group D (7/400, 1.8%), group F (5/400, 1.3%), group C (4/400, 1.0%), and clade I (2/400, 0.5%). In addition, 87 isolates (21.8%) could not be assigned to any phylogenetic group. S1 Fig shows a representative agarose gel of *E. coli* phylogenetic grouping.

### Screening and confirmation of *E. coli* ST131

Among the 192 phylogroup B2 isolates, 26 (13.5%) were positive for ST131-associated SNP markers. Most of these isolates were recovered from dogs (22/26, 84.6%), whereas four isolates (15.4%) were obtained from cats. Multilocus sequence typing confirmed that all 26 putative isolates belonged to sequence type 131. Seven isolates had been included in previous studies investigating ESBL-producing *E. coli* [14]; therefore, the remaining 19 ST131 isolates were selected for further characterization in the present study.

### Detection of virulence genes

All 19 ST131 isolates carried *fyuA* (19/19, 100.0%). None were positive for *focG* (0/19, 0.0%). The prevalence of the remaining virulence genes was as follows: *traT* (18/19, 94.7%), *iutA* (17/19, 89.5%), *kpsMT* II (16/19, 84.2%), *K5* (9/19, 47.4%), *papAH* (5/19, 26.3%), *cnf1* (3/19, 15.8%), *ibeA* (3/19, 15.8%), *K1* (2/19, 10.5%), *hlyA* (2/19, 10.5%), and *sfa/focDE* (1/19, 5.3%). Detailed virulence profiles of each isolate are presented in Table 1.

**Table 1:** Characteristics of *E. coli* ST131 isolates from dogs and cats with urinary tract infections, including serotypes, virulence genes, and antimicrobial resistance profiles.

Case No.	Species	Serotype	Virulence gene	Antibiotic resistance^a^
10	dog	O25b *fimH22*	*iutA*, *fyuA*, *K1*, *kpsMT* II, *ibeA*, *traT*	CTA CTF AMX AMP GEN TET CIP SXT
134	dog	O25b *fimH27*	*iutA*, *fyuA*, *kpsMT* II, *K5*, *traT*	CTA CTF AMX AMP GEN TET CIP ENR SXT
494	dog	O25b *fimH30*	*papAH, iutA*, *fyuA*, *cnf1*, *hlyA*, *kpsMT* II, *K5*, *traT*	CTA CTF AMX AMP GEN CIP ENR
646	dog	O25b *fimH22*	*papAH*, *sfa/focDE*, *fyuA*, *hlyA*, *ibeA*, *traT*	CTF AMX AMP GEN CIP SXT
668	dog	O25b *fimH30*	*iutA*, *fyuA*, *kpsMT* II, *K5*, *traT*	CTA CTF AMX AMP GEN CIP ENR SXT
881	dog	O25b *fimH30*	*iutA*, *fyuA*, *kpsMT* II, *K5*	CTA CTF AMX AMP GEN CIP ENR
1303	dog	O16 *fimH41*	*fyuA*, *traT*	CTA CTF AMX AMP GEN TET CIP SXT
1444	dog	O25b *fimH30*	*iutA*, *fyuA*, *kpsMT* II, *K5*, *traT*	CTA CTF AMX AMP TET CIP ENR SXT
2023	dog	O25b *fimH22*	*iutA*, *fyuA*, *kpsMT* II, *K5*, *traT*	CTF AMX AMP CIP ENR
2581	dog	O25b *fimH30*	*iutA*, *fyuA*, *kpsMT* II, *K5*, *traT*	CTA CTF AMX AMP GEN TET CIP ENR
2653	dog	O16 *fimH41*	*iutA*, *fyuA*, *kpsMT* II, *K5*, *traT*	CTF AMX AMP GEN CIP ENR
2684	dog	O25b *fimH30*	*iutA*, *fyuA*, *kpsMT* II, *K5*, *traT*	CTA CTF AMX AMP GEN TET CIP ENR SXT
2972	dog	O16 *fimH41*	*papAH*, *iutA*, *fyuA*, *cnf1*, *kpsMT* II, *traT*	CTA CTF AMX AMP GEN TET CIP ENR SXT
2995	dog	O16 *fimH41*	*papAH*, *iutA*, *fyuA*, *cnf1*, *kpsMT* II, *traT*	CTA CTF AMX AMP GEN TET CIP SXT
3004	dog	O16 *fimH41*	*fyuA*, *kpsMT* II, *traT*	CTA AMX AMP GEN CIP SXT
268	cat	O16 *fimH41*	*papAH*, *iutA*, *fyuA*, *K1*, *kpsMT* II, *ibeA*, *traT*	CTA CTF AMX AMP CIP
747	cat	O16 *fimH41*	*iutA*, *fyuA*, *kpsMT* II, *traT*	CTA CTF AMX AMP TET CIP SXT
2118	cat	O16 *fimH41*	*iutA*, *fyuA*, *kpsMT* II, *traT*	CTF AMX AMP
2311	cat	O16 *fimH41*	*iutA*, *fyuA*, *traT*	CTF AMX AMP GEN TET CIP ENR SXT

a: antimicrobials. Cefotaxime: CTA. Ceftiofur: CTF. Meropenem: MER. Amoxicillin: AMX. Ampicillin: AMP. Gentamicin: GEN. Tetracycline: TET. Ciprofloxacin: CIP: Enrofloxacin: ENR. Trimethoprim–sulfamethoxazole: SXT.

### Antimicrobial susceptibility test

All ST131 isolates were resistant to amoxicillin and ampicillin, whereas all remained susceptible to meropenem. Resistance rates for the other tested antimicrobials were as follows: ceftiofur and ciprofloxacin (95%), cefotaxime and gentamicin (74%), trimethoprim–sulfamethoxazole (63%), enrofloxacin (58%), and tetracycline (53%). The proportions of susceptible, intermediate, and resistant isolates for each antimicrobial agent are shown in S2 Fig. Individual isolate profiles are summarized in Table 1.

### O-antigen, *fimH* subclone, and plasmid replicon typing

Only two O-antigen types, O25b and O16, were identified among the ST131 isolates. Four *fimH* subtypes—*fimH22*, *fimH27*, *fimH30*, and *fimH41*—were detected. Notably, all O16 isolates belonged exclusively to the *fimH41* subtype (Table 1). Plasmid replicon typing identified multiple incompatibility groups, with detailed results summarized in Table 2.

**Table 2:** β-lactamase genes, transconjugation results, and plasmid replicon types of *E. coli* ST131 isolates.

Case No.	Species	*bla* in donor strain	*bla* in transconjugant	Replicon types	Plasmid family
10	dog	*bla*_CMY-178_	*bla*_CMY-178_	FIC, P, FIIA, FIB, I1, HI1, N	F, P, I1, HI1, N
134	dog			B/O, FIC, FIB, I1	B/O, F, I1
494	dog	*bla*_CTX-M-55 + CTX-M-17 + TEM-102_	*bla*_CTX-M-17_	K/B, FIIA, FIA, FIB	K, FII, F
646	dog			B/O, FIC, K/B, FIIA, FIB	B/O, K, F
668	dog	*bla*_CTX-M-55 + CTX-M-27 + TEM-12_	*bla*_CTX-M-27_	K/B, FIIA, FIA, FIB	K, F
881	dog			B/O, FIC, FIIA, FIA, FIB, Y	B/O, F, Y
1303	dog			B/O, FIC, P, FIIA, FIB	B/O, F, P
1444	dog			B/O, FIC, A/C, P, T, FIIA, FIA, FIB	B/O, F, A/C, P, T
2023	dog			FIA, FIB	F
2581	dog	*bla*_CTX-M-17_	*bla*_CTX-M-17_	B/O, FIC, P, K/B, FIIA, FIA, FIB	B/O, F, P, K
2653	dog			B/O, FIC, P, T, FIIA, FIA, FIB, L/M	B/O, F, P, T, L/M
2684	dog			B/O, P, T, K/B, FIIA, FIA, FIB	B/O, P, T, K, F
2972	dog	*bla*_CTX-M-27_	*bla*_CTX-M-27_	B/O, P, T, FIIA, FIB, I1	B/O, P, T, F, I1
2995	dog	*bla*_CTX-M-17_	*bla*_CTX-M-17_	B/O, FIC, P, T, FIIA, FIB	B/O, F, P, T
3004	dog			FIC, P, T, FIIA, FIB	F, P, T
268	cat			B/O, K/B, FIA, FIB, Frep	B/O, K, F, Frep
747	cat			B/O, A/C, FIIA, FIB	B/O, A/C, F
2118	cat			B/O, A/C, T, K/B, FIIA, FIB	B/O, A/C, T, K, F
2311	cat			B/O, FIIA, FIA, FIB	B/O, F

### Detection of β-lactamase genes and conjugation test

Among the 19 ST131 isolates, five carried ESBL genes and one carried an AmpC gene; all six isolates were recovered from dogs. The only AmpC gene detected was *bla*_CMY-178_ (case 10), which was successfully transferred to the *E. coli* J53 recipient strain. The ESBL-producing isolates carried *bla*_CTX-M-1_-group (CTX-M-55), *bla*_CTX-M-9_-group (CTX-M-17 and CTX-M-27), and *bla*_TEM_ group genes (TEM-12 and TEM-102). Notably, only *bla*_CTX-M-9_-group genes were successfully transferred in conjugation experiments, whereas *bla*_CTX-M-55_ and *bla*_TEM_ variants were not transferred. Detailed results for donor strains, transconjugants, and plasmid replicon types are presented in Table 2.

## Discussion

This study identified *E. coli* ST131 in 6.5% of urinary *E. coli* isolates recovered from companion animals in Taiwan. This prevalence was higher than that reported in previous studies from Germany (3.5%) [34] and from healthy dogs in Taiwan (2.3%) [13]. The higher prevalence observed in the present study may reflect differences in sampling populations, antimicrobial exposure, or regional epidemiological patterns.

Among the virulence-associated genes examined, *fyuA* was the most frequently detected and was present in all ST131 isolates. The *fyuA* gene encodes the receptor of the yersiniabactin iron-acquisition system, which enhances bacterial fitness in iron-limited host environments and has been widely associated with uropathogenic *E. coli* [35]. The distribution of virulence genes in ST131 isolates appears to vary geographically and across host species. Previous studies in Europe reported associations between companion-animal ST131 isolates and *ibeA* or *kpsMT* II, whereas only *kpsMT* II was highly prevalent in the present study and *ibeA* was detected less frequently [34]. Another study reported no significant correlations among the virulence genes *traT*, *cnf1*, and *fyuA* [34]. However, the detection rates of *fyuA* and *traT* in the present ST131 isolates were notably high—100.0% (19/19) and 94.8% (18/19), respectively. These findings suggest that the prevalence of virulence genes in ST131 isolates may vary across geographic regions. The prevalence of virulence genes in ST131 isolates also varies across studies. For example, in a study conducted in Iran, *fyuA* was detected in all ST131 isolates (100.0%), consistent with the findings of the present study. The detection rates of other genes—such as *ibeA* (1.4%), *traT* (95.7%), and *K5* (49.3%)—were also similar to those observed in the present study. However, the detection rates of *cnf1* (26.1%) and *hlyA* (29.0%) were higher than those reported in the present study [36].

The distribution and detection rates of ESBL and AmpC beta-lactamase genotypes in ST131 isolates also vary globally. Studies have shown that in most Asian countries—including Taiwan, China, and Japan, but excluding India—*bla*_CTX-M-14_ of the CTX-M-9 group is the most common ESBL genotype in ST131 isolates. *bla*_CTX-M-15_ of the CTX-M-1 group is also prevalent in ST131 isolates in Japan but relatively rare in those from Indonesia and China [37]. However, although *bla*_CTX-M-15_, which is predominantly detected in Europe, was not identified in the ST131 isolates analyzed in this study, the most frequently detected ESBL genes were *bla*_CTX-M-17_ (*n* = 3) and *bla*_CTX-M-27_ (*n* = 2), both belonging to the CTX-M-9 group. One strain carrying *bla*_CTX-M-17_ and another carrying *bla*_CTX-M-27_ also harbored *bla*_CTX-M-55_, a genotype belonging to the CTX-M-1 group. *bla*_CTX-M-55_ has been widely detected in food-producing and companion animals in China, and its geographic distribution is largely concentrated in Asia [27,38,39]. A study conducted in the United Kingdom reported that the detection rate of *bla*_CTX-M-15_, previously the dominant ESBL variant in Europe, has declined and has been increasingly replaced by *bla*_CTX-M-55_ [40]. Among the AmpC-related genotypes, *bla*_CMY-178_, a novel variant of *bla*_CMY_, was the only one identified in this study (carried by ST131 case 10). In both human and animal specimens, *bla*_CMY_-related genotypes are frequently detected in *E. coli*, with *bla*_CMY-2_ being the most prevalent variant. These genes are commonly located on IncA/C and IncI plasmids [41,42]. Studies have shown that *bla*_CMY-178_ confers higher resistance to ceftazidime–avibactam (CAZ–AVI) than *bla*_CMY-172_, another CAZ–AVI resistance gene [43].

In the conjugation test conducted in this study, ESBL/AmpC-producing strains carrying a single β-lactamase gene successfully transferred the gene to the recipient strain. In contrast, two strains harboring multiple β-lactamase genes (494 and 668) each transferred only one. Specifically, strain 494 (*bla*_CTX-M-55+CTX-M-17+TEM-102_) transferred only *bla*_CTX-M-17_, and strain 668 (*bla*_CTX-M-55+CTX-M-27+TEM-12_) transferred only *bla*_CTX-M-27_, both of which belong to the CTX-M-9 group. A possible explanation for this selective transfer is that the β-lactamase genes are located on different plasmids. Previous studies have further indicated that certain ESBL and AmpC genotypes are strongly associated with specific plasmid types [44–46]. This may indicate that different genes were located on distinct plasmids with variable transfer efficiencies, or that transfer conditions were suboptimal for certain plasmid types. Therefore, failure of transfer in vitro should not be interpreted as evidence of non-transferability in natural settings.

Among the ST131 isolates examined in this study, IncF (19/19, 100.0%) and IncB/O (15/19, 78.9%) were the most frequently detected plasmid types. IncF plasmids were identified in all ST131 isolates, consistent with previous reports showing strong associations between ST131 and IncF plasmid lineages [47]. These plasmids commonly carry antimicrobial resistance determinants and virulence-associated genes, thereby contributing to the successful global dissemination of ST131 [48,49].

A proportion of isolates could not be assigned to a phylogenetic group using the Clermont quadruplex PCR method. This may be attributable to sequence variation in primer-binding regions, inherent limitations of the PCR-based typing scheme, or variation in DNA quality among archived isolates. Future whole-genome sequencing studies may improve phylogenetic resolution. Capsule-associated virulence factors, including K1, have been reported in certain extraintestinal pathogenic *E. coli* (ExPEC) lineages, particularly those associated with invasive infections such as neonatal meningitis [5,20]. However, previous studies of ST131 have shown considerable variability in virulence gene profiles across subclones and host sources [9,31], and a consistent association between specific *fimH* subclones, including *fimH22*, and K1 capsule has not been clearly established. In the present study, only one of the three *fimH22* isolates was K1-positive by PCR, suggesting heterogeneity among companion-animal-derived ST131 isolates. This variation may reflect differences in genetic background, host adaptation, or selective pressures across ecological niches.

This study has several limitations. First, the isolates were obtained from a single veterinary teaching hospital and may not fully represent the broader companion-animal population in Taiwan. Second, the number of confirmed ST131 isolates available for detailed characterization was limited. Third, whole-genome sequencing was not performed and could provide additional insights into clonality, resistance gene context, and transmission dynamics. Despite these limitations, the present study provides valuable baseline data on the prevalence, resistance characteristics, and virulence-associated profiles of *E. coli* ST131 from dogs and cats with urinary tract infections in Taiwan. Continued surveillance of this high-risk lineage is warranted under a One Health framework.

## Conclusion

This study demonstrated the presence of *E. coli* ST131 among dogs and cats with urinary tract infections in Taiwan. Most isolates exhibited multidrug resistance and carried multiple virulence-associated genes, highlighting the clinical relevance of this lineage in companion animals. The detection of transferable β-lactamase genes and globally recognized ST131 subclones further underscores the potential public health importance of these isolates. Continued molecular surveillance and prudent antimicrobial stewardship in veterinary medicine are warranted within a One Health framework.

## Supporting information

S1 Table Primers used for *E. coli* phylogenetic grouping. (DOCX)

S2 Table Primers used for MLST analysis. (DOCX)

S3 Table Primers used for virulence gene detection. (DOCX)

S4 Table Primers used for detecting β-lactamase genes in *E. coli.* (DOCX)

S5 Table Primers used for O-antigen and *fimH* subclone typing. (DOCX)

S6 Table Primers used for plasmid replicon typing. (DOCX)

S1 Fig Quadruplex PCR profiles for Clermont phylogenetic grouping. (JPG)

S2 Fig Antimicrobial susceptibility profiles of *E. coli* ST131 isolates (n = 19). (JPG)
