# Supplementary material for: Characteristics of Escherichia coli ST131 strains isolated from dogs and cats with urinary tract infections in a teaching hospital in Taiwan
Source: PLoS One. 2026 May 22;21(5):e0350088. doi: 10.1371/journal.pone.0350088 (PMC13196923; doi:10.1371/journal.pone.0350088)
Supplement: S1 Table — (DOCX) [file pone.0350088.s001.docx]

S1 Table. Primers used for *E. coli* phylogenetic grouping

| PCR target | Primer | Sequence (5’-3’) | Annealing temperature (^o^C) | Predicted PCR size (bp) | References |
| --- | --- | --- | --- | --- | --- |
| *chuA* | chuA-F | ATGGTACCGGACGAACCAAC | 59 | 288 | [17] |
|  | chuA-R | TGCCGCCAGTACCAAAGACA |  |  |  |
| *yjaA* | yjaA-F | CAAACATGAAGTGTCAGGAG | 59 | 211 | [17] |
|  | yjaA-R | AATGCGTTCCTCAACCTGTG |  |  |  |
| *TspE4.C2* | TspE4.C2-F | CACTATTCGTAAGGTCATCC | 59 | 152 | [17] |
|  | TspE4.C2-R | AGTTTATCGCTGCGGGTCGC |  |  |  |
| *arpA* | arpA-F | AACGCTATTCGCCAGCTTGC | 59 | 400 | [17] |
|  | arpA-R | TCTCCCCATACCGTACGCTA |  |  |  |
