# Supplementary material for: Characteristics of Escherichia coli ST131 strains isolated from dogs and cats with urinary tract infections in a teaching hospital in Taiwan
Source: PLoS One. 2026 May 22;21(5):e0350088. doi: 10.1371/journal.pone.0350088 (PMC13196923; doi:10.1371/journal.pone.0350088)
Supplement: S2 Table — (DOCX) [file pone.0350088.s002.docx]

S2 Table. Primers used for MLST analysis

| PCR target | Primer | Sequence (5’-3’) | Annealing Temperature (^o^C) | Predicted PCR size (bp) | Reference |
| --- | --- | --- | --- | --- | --- |
| *adk* | adk-F | ATTCTGCTTGGCGCTCCGGG | 54 | 583 | [19] |
|  | adk-R | CCGTCAACTTTCGCGTATTT |  |  |  |
| *fumC* | fumC-F | TCACAGGTCGCCAGCGCTTC | 54 | 806 | [19] |
|  | fumC-R | GTACGCAGCGAAAAAGATTC |  |  |  |
| *gyrB* | gyrB-F | TCGGCGACACGGATGACGGC | 60 | 911 | [19] |
|  | gyrB-R | ATCAGGCCTTCACGCGCATC |  |  |  |
| *icd* | icd-F | ATGGAAAGTAAAGTAGTTGTTCCGGCACA | 54 | 878 | [19] |
|  | icd-R | GGACGCAGCAGGATCTGTT |  |  |  |
| *mdh* | mdh-F | ATGAAAGTCGCAGTCCTCGGCGCTGCTGGCGG | 60 | 932 | [19] |
|  | mdh-R | TTAACGAACTCCTGCCCCAGAGCGATATCTTTCTT |  |  |  |
| *purA* | purA-F | CGCGCTGATGAAAGAGATGA | 54 | 816 | [19] |
|  | purA-R | CATACGGTAAGCCACGCAGA |  |  |  |
| *recA* | recA-F | CGCATTCGCTTTACCCTGACC | 58 | 780 | [19] |
|  | recA-R | TCGTCGAAATCTACGGACCGGA |  |  |  |
