# Supplementary material for: Characteristics of Escherichia coli ST131 strains isolated from dogs and cats with urinary tract infections in a teaching hospital in Taiwan
Source: PLoS One. 2026 May 22;21(5):e0350088. doi: 10.1371/journal.pone.0350088 (PMC13196923; doi:10.1371/journal.pone.0350088)
Supplement: S3 Table — (DOCX) [file pone.0350088.s003.docx]

## S3 Table. Primers used for virulence gene detection

| PCR target | Primer | Sequence (5’-3’) | | Annealing temperature (^o^C) | Predicted PCR size (bp) | References |
| --- | --- | --- | --- | --- | --- | --- |
| *papAH* | papAH-F | ATGGCAGTGGTGTCTTTTGGTG | 63 | | 720 | [20] |
|  | papAH-R | CGTCCCACCATACGTGCTCTTC |  |  |  |  |
| *sfa/ focDE* | sfa/ focDE-F | CTCCGGAGAACTGGGTGCATCTTAC | 63 | | 410 | [20] |
|  | sfa/ focDE-R | CGGAGGAGTAATTACAAACCTGGCA |  |  |  |  |
| *focG* | focG-F | CAGCACAGGCAGTGGATACGA | 63 | | 360 | [20] |
|  | focG-R | GAATGTCGCCTGCCCATTGCT |  |  |  |  |
| *iutA* | iutA-F | GGCTGGACATCATGGGAACTGG | 63 | | 300 | [20] |
|  | iutA-R | CGTCGGGAACGGGTAGATCG |  |  |  |  |
| *fyuA* | fyuA-F | TGATTAACCCCGCGACGGGAA | 63 | | 880 | [20] |
|  | fyuA-R | CGCAGTAGGCACGATGTTGTA |  |  |  |  |
| *cnf1* | cnf1-F | AAGATGGAGTTTCCTATGCAGGAG | 63 | | 498 | [20] |
|  | cnf1-R | CATTCAGAGTCCTGCCCTCATTATT |  |  |  |  |
| *hlyA* | hlyA-F | AACAAGGATAAGCACTGTTCTGGCT | 63 | | 1177 | [20] |
|  | hlyA-R | ACCATATAAGCGGTCATTCCCGTCA |  |  |  |  |
| *K1* | K1-F | TAGCAAACGTTCTATTGGTGC | 63 | | 153 | [20] |
|  | K1-R | CATCCAGACGATAAGCATGAGCA |  |  |  |  |
| *kpsMT* II | kpsMT II-F | GCGCATTTGCTGATACTGTTG | 63 | | 272 | [20] |
|  | kpsMT II-R | CATCCAGACGATAAGCATGAGCA |  |  |  |  |
| *K5* | K5-F | CAGTATCAGCAATCGTTCTGTA | 63 | | 159 | [20] |
|  | K5-R | CATCCAGACGATAAGCATGAGCA |  |  |  |  |
| *ibeA* | ibeA-F | AGGCAGGTGTGCGCCGCGTAC | 63 | | 170 | [20] |
|  | ibeA-R | TGGTGCTCCGGCAAACCATGC |  |  |  |  |
| *traT* | traT-F | GGTGTGGTGCGATGAGCACAG | 63 | | 290 | [20] |
|  | traT-R | CACGGTTCAGCCATCCCTGAG |  |  |  |  |
