# Supplementary material for: Characteristics of Escherichia coli ST131 strains isolated from dogs and cats with urinary tract infections in a teaching hospital in Taiwan
Source: PLoS One. 2026 May 22;21(5):e0350088. doi: 10.1371/journal.pone.0350088 (PMC13196923; doi:10.1371/journal.pone.0350088)
Supplement: S4 Table — (DOCX) [file pone.0350088.s004.docx]

S4 Table. Primers used for detecting β-lactamase genes in *E. coli*

| PCR target | Primer | Sequence (5’-3’) | Annealing temperature (^o^C) | Predicted PCR size (bp) | References |
| --- | --- | --- | --- | --- | --- |
| *bla*_TEM_ | TEM-F | TCGGGGAAATGTGCGCG | 55 | 972 | [23] |
|  | TEM-R | TGCTTAATCAGTGAGGCACC |  |  |  |
| *bla*_SHV_ | SHV-F | GCCTTTATCGGCCCTCATCAA | 54 | 819 | [24] |
|  | SHV-R | TCCCGCAGATAAATCACCACAATG |  |  |  |
| *bla*_CTX-M-1_ | CTX-M-1-F | CCCATGGTTAAAAAATCACTGC | 54 | 942 | [25] |
|  | CTX-M-1-R | CAGCGCTTTGCCGTCTAAG |  |  |  |
| *bla*_CTX-M-2_ | CTX-M-2-F | CGACGCTACCCCTGCTATT | 52 | 552 | [26] |
|  | CTX-M-2-R | CCAGCGTCAGATTTTTCAGG |  |  |  |
| *bla*_CTX-M-8_ | CTX-M-8-F | TCGCGTTAAGCGGATGATGC | 52 | 666 | [26] |
|  | CTX-M-8-R | AACCCACGATGTGGGTAGC |  |  |  |
| *bla*_CTX-M-9_ | CTX-M-9-F | ATGGTGACAAAGAGAGTGCAAC | 55 | 876 | [27] |
|  | CTX-M-9-R | TTACAGCCCTTCGGCGATGATT |  |  |  |
| *bla*_CTX-M-25_ | CTX-M-25-F | GCACGATGACATTCGGG | 52 | 327 | [26] |
|  | CTX-M-25-R | AACCCACGATGTGGGTAGC |  |  |  |
| *bla*_CIT_ | CIT-M-F | TGGCCAGAACTGACAGGCAAA | 64 | 462 | [28] |
|  | CIT-M-R | TTTCTCCTGAACGTCGCTGGC |  |  |  |
| *bla*_MOX_ | MOX-M-F | GCTGCTCAAGGAGCACAGGAT | 64 | 520 | [28] |
|  | MOX-M-R | CACATTGACATAGGTGTGGTGC |  |  |  |
| *bla*_DHA_ | DHA-M-F | AACTTTCACAGCTGTGCTGGGT | 64 | 405 | [28] |
|  | DHA-M-R | CCGTACGCATACTGGCTTTGC |  |  |  |
| *bla*_CMY_ | CMY-M-F | ATGATGAAAAAATCGTTATGCT | 64 | 1146 | [28] |
|  | CMY-M-R | TTATTGCAGCTTTTCAAGAATGCG |  |  |  |
| *bla*_EBC_ | EBC-M-F | TCGGTAAAGCCGATGTTGCGG | 64 | 302 | [28] |
|  | EBC-M-R | CTTCCACTGCGGCTGCCAGTT |  |  |  |
| *bla*_FOX_ | FOX-M-F | AACATGGGGTATCAGGGAGATG | 64 | 190 | [28] |
|  | FOX-M-R | CAAAGCGCGTAACCGGATTGG |  |  |  |
