# Supplementary material for: Characteristics of Escherichia coli ST131 strains isolated from dogs and cats with urinary tract infections in a teaching hospital in Taiwan
Source: PLoS One. 2026 May 22;21(5):e0350088. doi: 10.1371/journal.pone.0350088 (PMC13196923; doi:10.1371/journal.pone.0350088)
Supplement: S5 Table — (DOCX) [file pone.0350088.s005.docx]

S5 Table. Primers used for O-antigen and *fimH* subclone typing

| PCR target | Primer | Sequence (5’-3’) | Annealing temperature (^o^C) | Predicted PCR size (bp) | References |
| --- | --- | --- | --- | --- | --- |
| *pabB* | O25b-F | TCCAGCAGGTGCTGGATCGT | 58 | 347 | [29] |
|  | O25b-R | GCGAAATTTTTCGCCGTACTGT |  |  |  |
| *trpA* | O16-F | AAAACCGCGCCGCGTTACCT | 58 | 145 | [30] |
|  | O16-R | CCAGAAATCGCGCCCGCATT |  |  |  |
| *fimH30* | fimH30*-*F | GCCAATGGTACCGCTATT | 49.2 | 500 | [31] |
|  | fimH30-R | GCTTTAATCGCCACCCCA |  |  |  |
| *ybbw* | fimH30Rx-F | GTTGCGGTCTGGGCA | 50 | 188 | [31] |
|  | fimH30Rx-R | TCCAGCACGTTCCAGGTG |  |  |  |
| *fimH35* | fimH35-F | CTGTAAAACCGCCAATGGTACA | 54.2 | 500 | [31] |
|  | fimH35-R | TGACATCACGAGCAGAAACATCG |  |  |  |
| *fimH27* | fimH27-F | TGGGGCAAAACCTGGTCGTG | 55.6 | 417 | [31] |
|  | fimH27-R | TGACATCACGAGCAGAAACATCG |  |  |  |
| *fimH22* | fimH22-F | TGGGGCAAAACCTGGTCGTG | 54.9 | 279 | [31] |
|  | fimH22-R | CAGCTTTAATCGCCACTCCC |  |  |  |
| *fimH41* | fimH41-F | CTGTAAAACCGCCAATGGTACA | 54.5 | 92 | [31] |
|  | fimH41-R | TTTGCCCCACATTCACGGCG |  |  |  |
