# Supplementary material for: Characteristics of Escherichia coli ST131 strains isolated from dogs and cats with urinary tract infections in a teaching hospital in Taiwan
Source: PLoS One. 2026 May 22;21(5):e0350088. doi: 10.1371/journal.pone.0350088 (PMC13196923; doi:10.1371/journal.pone.0350088)
Supplement: S6 Table — (DOCX) [file pone.0350088.s006.docx]

S6 Table. Primers used for plasmid replicon typing

| PCR target | Primer | | Sequence (5’-3’) | Annealing temperature (^o^C) | Predicted PCR size (bp) | References |
| --- | --- | --- | --- | --- | --- | --- |
| B/O | B/O-F | GCGGTCCGGAAAGCCAGAAAAC | | 60 | 159 | [32] |
|  | B/O-R | TCTGCGTTCCGCCAAGTTCGA | |  |  |  |
| FIC | FIC-F | GTGAACTGGCAGATGAGGAAGG | | 60 | 262 | [32] |
|  | FIC-R | TTCTCCTCGTCGCCAAACTAGAT | |  |  |  |
| A/C | A/C-F | GAGAACCAAAGACAAAGACCTGGA | | 60 | 465 | [32] |
|  | A/C-R | ACGACAAACCTGAATTGCCTCCTT | |  |  |  |
| P | P-F | CTATGGCCCTGCAAACGCGCCAGAAA | | 60 | 534 | [32] |
|  | P-R | TCACGCGCCAGGGCGCAGCC | |  |  |  |
| T | T-F | TTGGCCTGTTTGTGCCTAAACCAT | | 60 | 750 | [32] |
|  | T-R | CGTTGATTACACTTAGCTTTGGAC | |  |  |  |
| K/B | K/B-F | GCGGTCCGGAAAGCCAGAAAAC | | 60 | 160 | [32] |
|  | K/B-R | TCTTTCACGAGCCCGCCAAA | |  |  |  |
| W | W-F | CCTAAGAACAACAAAGCCCCCG | | 60 | 242 | [32] |
|  | W-R | GGTGCGCGGCATAGAACCGT | |  |  |  |
| FIIA | FIIA-F | CTGTCGTAAGCTGATGGC | | 60 | 270 | [32] |
|  | FIIA-R | CTCTGCCACAAACTTCAGC | |  |  |  |
| FIA | FIA-F | CCATGCTGGTTCTAGAGAAGGTG | | 60 | 462 | [32] |
|  | FIA-R | GTATATCCTTACTGGCTTCCGCAG | |  |  |  |
| FIB | FIB-F | GGAGTTCTGACACACGATTTTCTG | | 60 | 702 | [32] |
|  | FIB-R | CTCCCGTCGCTTCAGGGCATT | |  |  |  |
| Y | Y-F | AATTCAAACAACACTGTGCAGCCTG | | 60 | 765 | [32] |
|  | Y-R | GCGAGAATGGACGATTACAAAACTTT | |  |  |  |
| I1 | I1-F | CGAAAGCCGGACGGCAGAA | | 60 | 139 | [32] |
|  | I1-R | TCGTCGTTCCGCCAAGTTCGT | |  |  |  |
| Frep | Frep-F | TGATCGTTTAAGGAATTTTG | | 60 | 270 | [32] |
|  | Frep-R | GAAGATCAGTCACACCATCC | |  |  |  |
| X | X-F | AACCTTAGAGGCTATTTAAGTTGCTGAT | | 60 | 376 | [32] |
|  | X-R | TGAGAGTCAATTTTTATCTCATGTTTTAGC | |  |  |  |
| HI1 | HI1-F | GGAGCGATGGATTACTTCAGTAC | | 60 | 471 | [32] |
|  | HI1-R | TGCCGTTTCACCTCGTGAGTA | |  |  |  |
| N | N-F | GTCTAACGAGCTTACCGAAG | | 60 | 559 | [32] |
|  | N-R | GTTTCAACTCTGCCAAGTTC | |  |  |  |
| HI2 | HI2-F | TTTCTCCTGAGTCACCTGTTAACAC | | 60 | 644 | [32] |
|  | HI2-R | GGCTCACTACCGTTGTCATCCT | |  |  |  |
| L/M | L/M-F | GGATGAAAACTATCAGCATCTGAAG | | 60 | 785 | [32] |
|  | L/M-R | CTGCAGGGGCGATTCTTTAGG | |  |  |  |
