# Supplementary figures and images for: Characteristics of Escherichia coli ST131 strains isolated from dogs and cats with urinary tract infections in a teaching hospital in Taiwan
Source: PLoS One. 2026 May 22;21(5):e0350088. doi: 10.1371/journal.pone.0350088 (PMC13196923; doi:10.1371/journal.pone.0350088)

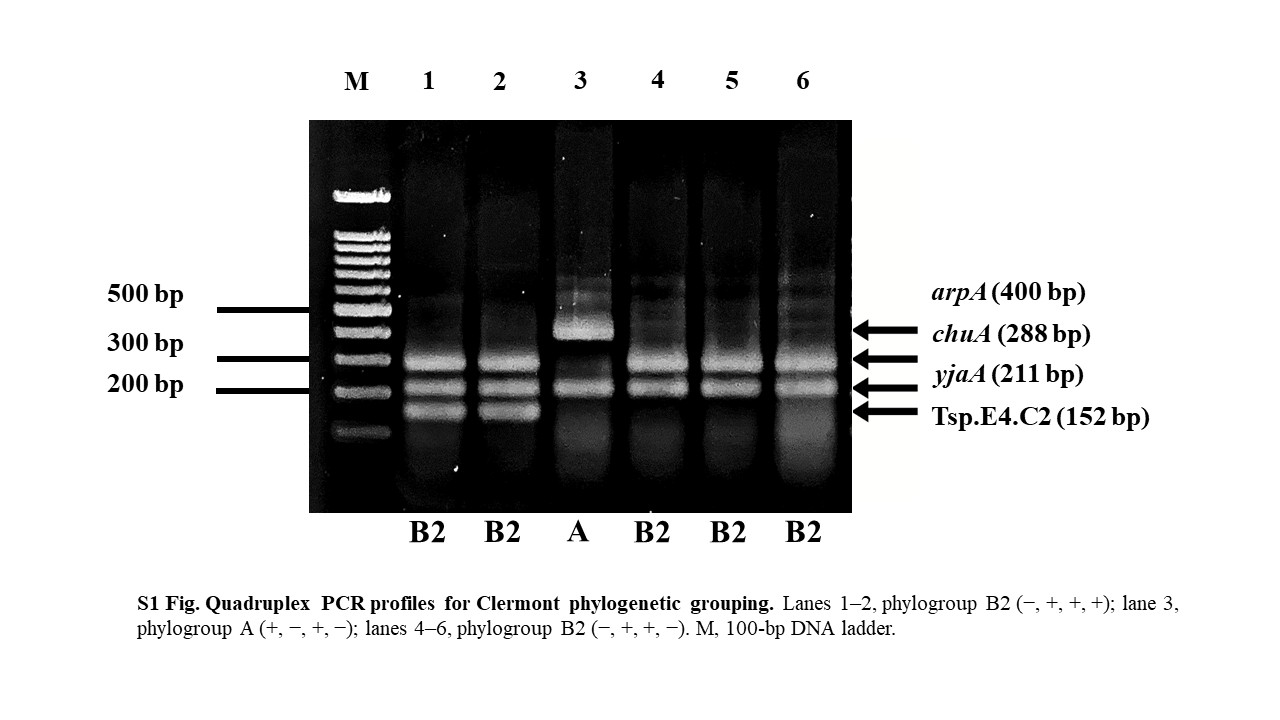

Supplement: S1 Fig — (JPG) [file pone.0350088.s007.jpg]

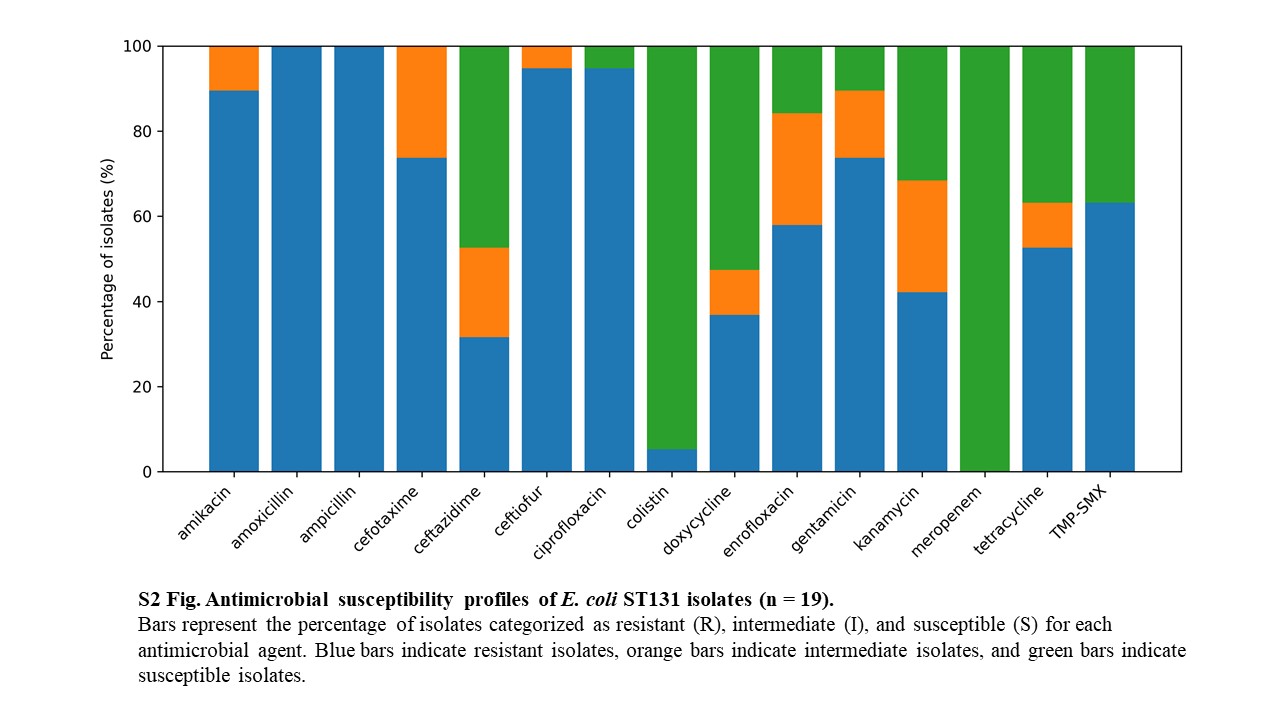

Supplement: S2 Fig — (JPG) [file pone.0350088.s008.jpg]
